# From COVID-19 research to vaccine application: why might it take 17 months not 17 years and what are the wider lessons?

**DOI:** 10.1186/s12961-020-00571-3

**Published:** 2020-06-08

**Authors:** Stephen R. Hanney, Steven Wooding, Jon Sussex, Jonathan Grant

**Affiliations:** 1 Health Economics Research Group, Brunel University London, Kingston Lane, Uxbridge, UK; 2 Research Strategy Office, University of Cambridge, Old Schools, Cambridge, UK; 3 RAND Europe, Westbrook Centre, Milton Road, Cambridge, UK; 4 Policy Institute at King’s, King’s College London, Virginia Woolf Building, 22 Kingsway, London, UK

**Keywords:** COVID-19, Coronavirus disease, SARS-CoV-2, Vaccine, Time-lags, Research translation, Matrix, Pandemic, Trials, Timescales, World Health Organization

## Abstract

It is often said that it takes 17 years to move medical research from bench to bedside. In a coronavirus disease (COVID-19) world, such time-lags feel intolerable. In these extraordinary circumstances could years be made into months? If so, could those lessons be used to accelerate medical research when the crisis eases?

To measure time-lags in health and biomedical research as well as to identify ways of reducing them, we developed and published (in 2015) a matrix consisting of overlapping tracks (or stages/phases) in the translation from discovery research to developed products, policies and practice. The matrix aids analysis by highlighting the time and actions required to develop research (and its translation) both (1) along each track and (2) from one track to another, e.g. from the discovery track to the research-in-humans track. We noted four main approaches to reducing time-lags, namely increasing resources, working in parallel, starting or working at risk, and improving processes.

Examining these approaches alongside the matrix helps interpret the enormous global effort to develop a vaccine for the 2019 novel coronavirus SARS-CoV-2, the causative agent of COVID-19. Rapid progress in the discovery/basic and human research tracks is being made through a combination of large-scale funding, work being conducted in parallel (between different teams globally and through working in overlapping tracks), working at greater (but proportionate) risk to safety than usual, and adopting various new processes. The overlapping work of some of the teams involves continuing animal research whilst entering vaccine candidates into Phase I trials alongside planning their Phase II trials. The additional funding available helps to reduce some of the usual financial risks in moving so quickly. Going forward through the increasingly large human trials for safety, dosage and efficacy, it will be vital to overlap work in parallel in the often challenging public policy and clinical tracks. Thus, regulatory and reimbursement bodies are beginning and preparing rapid action to pull vaccines proving to be safe and effective through to extraordinarily rapid application to the general population. Monitoring the development of a COVID-19 vaccine using the matrix (modified as necessary) could help identify which of the approaches speeding development and deployment could be usefully applied more widely in the future.

## Understanding the time-lags between research and its application

The idea that it takes, on average, 17 years from starting research to its translation into products, policies and practice has gained considerable traction in recent years since the review by Morris, Wooding and Grant asked: “*The answer is 17 years, what is the question?*” [1]. However, with coronavirus disease (COVID-19) causing so many excess deaths and effectively shutting down many usual social and economic activities, a 17-year wait for a vaccine feels intolerable. So how could the lag between bench and bedside be reduced to, say, just 17 months (or even less)? In an extreme crisis, extraordinary things can be achieved, yet there are reasons for the 17-year time-lag – so what has changed?

The 17-year time-lag quoted by Morris et al. was deduced from a review of 23 papers; however, as the authors concluded, different studies were “[u]*sing different endpoints, different domains and different approaches*” [1]. The authors identified a group of studies that examined a broad range of interventions from the start of research to some aspect of adoption in clinical practice [2–5]. These studies coalesced around an average figure of 17 years, which has since been commonly cited in the policy debate and literature [6–8].

Buxton et al.’s study of the returns to United Kingdom public and charitably funded cardiovascular disease research [2] was one of the papers included in Morris et al.’s review [1]. Despite the 17-year time-lag estimated in their study, Buxton et al. [2] calculated that there was nevertheless a high rate of return on medical research, which included both the value of improved health and the value to the economy. (Subsequent to the publication of Morris et al.’s review [1], Buxton et al. found similar timelines in their further studies of the returns to research on musculoskeletal disease and cancer [8, 9].

**Fig. 1:**
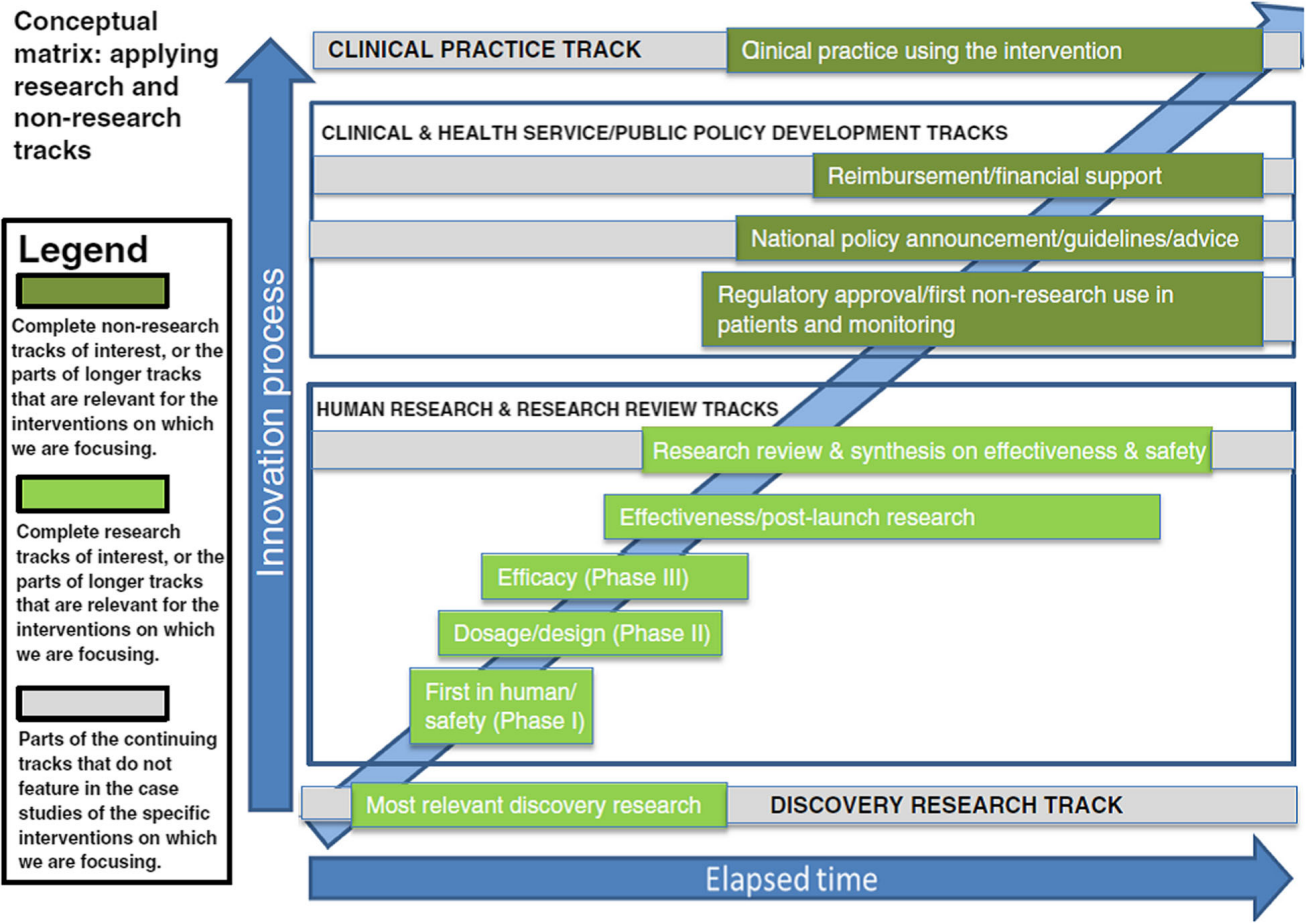
Conceptual matrix for measuring and understanding time-lags Source: Hanney et al. [10]

The rate of return, however, would have been even higher if the time-lags had been shorter. This highlighted the importance of understanding time-lags and how they could be reduced. Subsequently, the United Kingdom’s Medical Research Council funded the authors of this Commentary, along with other authors, to explore this issue. In 2015, we published our findings including a conceptual matrix that informed our analysis (Fig. 1) [10]. That matrix, along with the approaches we identified for reducing time-lags, provides a framework to examine the current race to develop a COVID-19 vaccine. Such analysis of the accelerated development, and its opportunity costs, could provide lessons both for accelerating the development of future therapies through to routine healthcare use and enhancing the approach used in future analyses of time-lags.

In the time-lags study, we developed an approach to present data on multiple tracks (or stages or phases) in the translation of research into healthcare improvements that went beyond a simple linear representation and recognised the importance of incorporating scope for overlaps. We noted the rich literature on the challenges faced in the diffusion of innovations [11], but focused on exploring the time-lags in the whole spectrum between early research and the eventual translation into health gains [10]. Our starting point was the process marker model developed by Trochim et al., which helpfully identified a series of “*operationally definable*” markers, or milestones, in the “*research –practice translation continuum*” [12]. We built on this considering each of the major steps in the translation process from early research through to adoption in the healthcare system. We built these markers into a matrix and tested the validity of our approach by identifying the markers in data from our previous analysis of the eventual healthcare impact in diverse countries resulting from a stream of animal and human research on the use of corticosteroids for the prevention of respiratory distress syndrome (RDS) [13]. This initial test showed promise and illustrated the value of featuring overlaps – in the RDS case, there was some adoption in clinical practice well before further activity had to return to the national policy and guidelines track to encourage wider adoption at an appropriate level in clinical practice.

The matrix consists of four main tracks, two of which contain the research (discovery research, which we defined very broadly, and human research/research review) and two of which cover the public policy and clinical practice developments. The two middle tracks each consist of a number of separate sub-tracks. The model facilitates the analysis of time-lags by allowing consideration of two linked issues: the time and actions required to move research (and its translation) (1) along any particular track and (2) from one track to another at the cross-over points between tracks. We conducted a series of seven case studies to test and refine the matrix. They covered diverse types of research, including examples of the development of drugs, screening, cognitive behavioural therapy and early intervention, in the two fields of cardiovascular disease and mental health [10]. These cases (along with the RDS example) highlighted the following:

- A time lapse between discovery and routine use is inevitable and desirable to ensure the safety, efficacy and cost-effectiveness of new interventions.
- Most timelines include some examples of where there had been considerable delays in the continuum as well as some examples of quite rapid movement from one track to the next (e.g. the 2008 policy to introduce national screening for abdominal aortic aneurysms in England was rapidly and comprehensively implemented in the clinical practice track to cover 300,000 people annually) [10].
- Using overlapping tracks facilitates the exploration of the time-lags, including in the later stages of the matrix involving public policy and widespread adoption in clinical practice – areas which the literature also shows are often subject to considerable delays [10, 11, 13, 14].

Our 2015 study also included an update of the Morris et al. review [1] and found a similar picture with identified papers not measuring time-lags in a comparable way, even though, in this instance, they predominantly had a pharmaceutical focus [10]. In these studies, there was more homogeneity about the end point than the start point for measuring but, because that common end point was the licensing/approval process, it meant that they were stopping the time-lag measurements much earlier than shown in our matrix.

### Approaches to reducing time-lags from early research to its translation

So, how might it be feasible to reduce 17 years to perhaps 17 months, or less? In diverse countries, there has been detailed analysis of various mechanisms for reducing elements of the time-lags, particularly ones focusing on parts of the continuum such as the track from evidence to practice and policy [14] and the regulatory approval and reimbursement tracks [15]. Below, however, we draw primarily on analysis in our own 2015 article [10] (and our dissemination of it [16]) to outline four overlapping approaches or strategies. In the subsequent section, we apply the approaches alongside the tracks in the matrix to explore the extraordinary efforts being made to reduce the elapsed time in the development of a vaccine for the 2019 novel coronavirus SARS-CoV-2, the causative agent of COVID-19.

#### Increasing resources

The case studies identified various situations in which translation would have been speeded up by more resources being available at key points in various tracks, including the resources to train additional cognitive behavioural therapists, which were eventually made available [10]. While very large sums are spent globally on medical research each year, the development of any specific intervention might be delayed because of insufficient resources or wastage. When detailed studies are conducted of the development of major new successful interventions along various tracks, it sometimes appears with hindsight that there were inexplicable delays at crucial points while decisions were made about whether to pursue the research. In reality, however, with so many new interventions always in the pipeline, at various points, queues develop for decisions, be it about funding of the next stage or track, or ethics approval, among others [10]. Queues could often be reduced by applying greater resources, which would also inevitably help facilitate some of the additional approaches below; however, increasing resources means, by definition, increased cost.

#### Working in parallel

A major advantage of creating the overlapping tracks in the matrix is that it promotes the idea that actions are taken in parallel at certain times. We reported an example of this happening with screening trials at the equivalent of the Phase II and Phase III trials [10]. When this happens, it can increase efficiency and reduce the time taken overall albeit likely at the cost of increasing risks – although it did not in this specific example. In addition to parallel work in different tracks of the matrix, there might also be coordinated parallel work by different research teams collaborating to speed up progress.

#### Starting or working at risk

The two key risks are safety risks and financial risks. Safety risks in the research processes might entail working in parallel in the next track. Safety risks might also involve liaison with the regulator about how far the Phase I-III trials have to go before the regulator will give approval for use. We identified the literature showing that drugs for HIV/AIDS had the shortest Phase III and overall times; sponsors were allowed to file New Drug Applications (NDAs) to the United States regulators without completing large-scale human trials [17]. We noted that “*the process seems to have been speeded up by the adoption by the regulator of a different benefit-risk profile in response to the particular circumstances posed by HIV/AIDS and the demands of patients*” [10]. In an additional file linked to our 2015 article, we also described the various steps that regulatory bodies, such as the Food and Drugs Administration in the United States, can take to speed up approval of a medication for serious diseases, especially where it is the first available treatment [10]. These include more frequent meetings and correspondence with the developers to discuss the collection of appropriate data and trial design. Furthermore, there can be a rolling review process where the parts of an NDA that are ready can be submitted instead of the usual procedure of waiting until the entire NDA is ready [10]. A framework for thinking about ways of accelerating this track was developed by one of the authors in work subsequent to our time-lags study [15]. The financial risks to the organisations developing drugs arise because trials in later phases of the human research track are larger and more expensive than the earlier ones. Furthermore, many candidate interventions in early trials are not successful [18]. For both these reasons pharmaceutical companies and other research organisations prefer to wait and confirm progress at one stage before moving onto the next [19]. However, in a crisis, companies might be willing to take more risk (and this is an example where the provision of additional resources could matter – see below).

#### Improving processes

This can apply along tracks as well as between tracks and an accumulation of small improvements to specific items, such as accelerated ethics and peer review, could add up to significant time reductions and a consequent improvement in efficiency in getting new interventions into use [10, 16]. In one case study, we noted that more resources would allow bodies making reimbursement decisions to more often adopt the speeded up process that the National Institute for Health and Care Excellence (NICE; England and Wales) used in the exceptional circumstances of a drug’s Phase IV trial being stopped early following significantly higher mortality rates in the trial’s other arm [10].

## Developing a COVID-19 vaccine in record time

None of the seven case studies in our study examined vaccine development and there are some features of that field that are likely to contribute to faster translation from research to application than in other fields. For example, in the past, the vaccine field has had relatively low market margins and is often seen as commercially unattractive [20–22]. As a consequence, quite a lot of the relevant research is conducted in universities, meaning that there are many ideas around the proof-of-concept stage that are ready to go – they provide a fertile area for integration once financial or other incentives appear. Nevertheless, the development of vaccines is still sometimes reported to take, on average, over 10 years and there is often an attrition rate for vaccine candidates of about 90% or higher as they go through the various tracks [18, 23, 24].

Despite those average figures, there are at least three particular features in the context of the development of a COVID-19 vaccine that are particularly important and play across various tracks, even if they are not likely to appear in many, or any, other situations. First, there is the intense and overwhelming nature of the COVID-19 crisis that is engulfing the globe and making it the top priority for action everywhere – meaning many prioritisation decisions are a given. Second, the widespread lockdowns and social distancing are also restricting research activities generally, with many laboratories not functioning apart from those conducting work linked to COVID-19 [22]. This highly unusual situation might mean that, in the short-term at least, concentrating resources on one area does not have the usual opportunity costs of depriving other areas of resources – because they have generally been put on hold. It is therefore possible that the queues for research resources will be much less in evidence. (However, the more usual pattern could rapidly reinsert itself as the lockdowns ease.)

Third, SARS-CoV-2 is the latest of a series of coronaviruses for which research teams have been seeking vaccines [18, 21]. Therefore, various tools, such as vaccine platforms that had already been developed or new ones that were in development, can be integrated in the search for a vaccine against the new disease. Linked to this, several non-market mechanisms were developed to make better preparation for a future epidemic, including the Coalition for Epidemic Preparedness Innovations (CEPI) based in Oslo, Norway. Established in 2017, it receives funding from governments and donors such as the Bill & Melinda Gates Foundation and the Wellcome Trust [21]. In addition to funding development of 17 vaccines against five priority pathogens, CEPI also “*funded programs for unknown emergent pathogens – programs for ‘Disease X’. ‘Disease X’ now has a name: COVID-19*” [21].

The four approaches above for speeding things up can now be considered, alongside the matrix in Fig. 1, to analyse how things are currently being accelerated. This draws on what has already been written about the extraordinary efforts being made by a global community of scientists, healthcare industries and coordinating organisations such as WHO and CEPI since a Chinese team published the genetic sequence of SARS-CoV-2 on 11 January 2020 [23]. Working with WHO, CEPI has developed and is “*continuously maintaining an overview of the global landscape of COVID-19 vaccine development*” [23]. CEPI has also estimated that many billions of dollars will be needed for the successful rapid development and manufacture of one or more vaccines [25].

When considering speeding up progress on developing and delivering a COVID-19 vaccine against in each of the four groups of tracks in Fig. 1, it is immediately obvious that the concept of overlapping tracks is hugely important and facilitates the idea of working in parallel on various items. Additionally, some of the drivers of the increased speed are the overlapping action in the regulatory and reimbursement tracks towards the top of the matrix, which can potentially play a role in pulling developments through more rapidly from the lower tracks.

The CEPI team have developed a paradigm specifically for a pandemic, which they contrast with a more linear traditional paradigm for vaccine development [19]. As with our Fig. 1, the key feature of this are the phases that overlap; additionally, it includes the development of manufacturing capacity in the overlapping phases, which will be crucial for the rapid mass production of the vaccines. We had not included manufacturing capacity in our matrix as investing in sufficient capacity is generally not the limiting factor in conventional development of new medicines. Consequently, manufacturing capacity did not arise as a delaying issue in our case studies – although there was possibly a somewhat comparable situation with the lack of capacity in the form of trained cognitive behavioural therapists, until resources were found for additional training. However, given the scale of manufacture likely to be necessary for COVID-19 vaccines and the hoped-for considerably shortened timescale, it is being highlighted as an issue in current discussions [25]. The analysis below linked to our matrix throws light on how events are unfolding in a timescale that is much more rapid than previously seen.

### Discovery research track

Partly through the large-scale funding provided through CEPI and others, some teams are reporting extremely rapid progress with basic preclinical/animal research; for example, a press release from Inovio, a United States company, on 6 April 2020 marking the launch of its Phase I clinical trial, stated the following: “*Preclinical data, which have been shared with global regulatory authorities and submitted as part of the IND* [Investigational New Drug] [application], *have shown promising immune response results across multiple animal models*” [26]. The company went on to say that additional preclinical studies “*will continue in parallel with the Phase 1 clinical trial*”. One of the keys to rapid progress, in this case with a DNA vaccine and in other cases, such as the University of Oxford’s Jenner Institute in the United Kingdom, with a vector vaccine, is that the teams had already been working on a vaccine for MERS-CoV, another coronavirus that is the causative agent for Middle East Respiratory Syndrome (MERS) [26, 27]. Gilbert explained how her team at the Jenner Institute had conducted research using improved research processes prior to the pandemic to create a new vector vaccine platform and had “*started thinking about an appropriate response to Disease X; how could we mobilise and focus our resources to go more quickly than we had ever gone before. And then Disease X arrived*” [27]. Gilbert is aiming to have a vaccine ready for use in Autumn 2020, a shorter timescale than any other team seems to have suggested; however, in terms of identifying the starting point of the relevant research, which is always problematic [10], her discovery research stretches back well before January 2020 [27]. A major new feature assisting the more rapid development of a vaccine now is the unprecedented level of global cooperation within the scientific community and with other relevant bodies, with, for example, scientists reporting important findings on conference calls organised by WHO rather than using time to write and publish academic papers [21, 22]. Some of the animal trials for both Inovio and the Jenner Institute are being conducted by a laboratory of Australia’s Commonwealth Scientific and Industrial Research Organisation. It pointed out that “*Normally it takes about one-to-two years to get to this point and we’ve in fact shortened that to a period of a couple of months*” [28]. Preclinical testing for the Jenner Institute has also been conducted in the United Kingdom and United States, with promising results reported [29].

Improved processes also have a role in reducing development time during the pandemic [21, 27]. In particular, novel platforms are being used, with those based on DNA or mRNA offering “*great flexibility in terms of antigen manipulation and potential for speed*” [23]. A partnership between another company, Moderna, and the United States National Institute of Allergy and Infectious Diseases (NIAID) started clinical testing of Moderna’s mRNA-based vaccine just 2 months after the announcement of the genetic sequence but initially skipped one of the stages of animal testing [28]. While the mRNA-based platform for delivering vaccines had been shown to be safe in humans, this COVID-19 vaccine had not [30], and “*mRNA-1273 faces numerous challenges in clinical development and manufacture before it has the possibility of being made available for global immunization*” [21]. Animal studies are usually a vital first step, required for regulatory approval.

### Human research and research review tracks

#### Phase I, II and III – first-in-human/safety, design/dosage, efficacy

We consider these three phases as one group because different approaches use somewhat different terminology but still cover the same essential items. The CEPI analysis of the COVID-19 vaccine development landscape published on 9 April 2020 reported that, of the 78 identified vaccine candidates confirmed as active, five were in Phase I trials – the studies by Moderna and Inovio described above, plus three Chinese studies [23]. Following links in that paper reveals that all five anticipate many months of research just for Phase I, although, as reported in late April 2020, CanSino, a Chinese company developing a vector vaccine [23], was the first to move into a, presumably overlapping, Phase II study [24]. In a press release on 30 April 2020, Inovio referred to potentially advancing “*to Phase 2/3 efficacy trials this summer*” [31]. Vital considerations in how far the candidate vaccines will move through the trial phases and the extent of overlap will be considerations of safety and financial risk. There are suggestions that some of the usual safety procedures that could constrain progress during the trials might be relaxed, with the NIAID, which is conducting the Phase I trial of Moderna’s mRNA vaccine, reportedly arguing that “*the risk of delaying the advancement of vaccines is much higher than the risk of causing illness in healthy volunteers*” [30]. Moderna’s Phase I trial initially enrolled 45 volunteers in the original three dose cohorts, but later added six more cohorts [32]. On 27 April 2020, Moderna, with funding from the United States Government’s Biomedical Advanced Research and Development Authority (BARDA), submitted an Investigational New Drug application to the Food and Drugs Administration for “*Phase 2 and late stage studies of mRNA-1273 if supported by safety data from the Phase 1 study*” and reported, on 1 May 2020, that it had received initial feedback on the design of a 600-participant Phase II trial, which it expected to start it in the second quarter of 2020, with a 12-month follow-up [32]. This again illustrates that regular liaison with the regulatory bodies is likely to be essential during the research as new platforms, new approaches and the immense urgency of the situation all come together.

Gilbert described the importance of work at the Jenner Institute running in parallel, funded by grants from various sources and, while the preclinical research was underway, she received ethical approval for the clinical trial and conditional approval from the United Kingdom’s regulatory authority to screen volunteers for trial enrolment [27]. Furthermore, in April 2020, drawing on the safety data from their previous trials of similar vaccines, the Jenner Institute team were able to discuss with the United Kingdom regulators the basis on which they could start a combined Phase II/Phase III trial with another 5000 participants [29] in addition to the 1102 in the Phase I trial [24]. Some of the grants received by this team also helped fund initial scaling up of vaccine production using facilities in the United Kingdom and elsewhere [27, 29]. The size and speed of the human trials that the Jenner Institute is progressing reflect the confidence gained from its prior research on other vaccines and animal testing of its COVID-19 vaccine in various models [27–29]. The apparently smaller and more gradual Phase I trial by Moderna, and plans for its Phase II trial, perhaps reflect the greater caution that is necessary in its human trials (despite its previous work on similar vaccines) as it avoided some of the animal trials prior to being the first to conduct human trials.

Mitigating the financial risk to running phases in parallel is the additional funding being devoted to developing a vaccine, with CEPI, among others, playing a key coordinating role in distributing donations. Also accelerating progress is that any decisions about a COVID-19 vaccine are going straight to the head of any queue for decisions about, for example, resources or ethics approval. In terms of encouraging the maximum participation by commercial enterprises, some of the debates being conducted in the ‘Reimbursement/financial support’ track could be extremely important in providing incentives.

#### Effectiveness/post-launch research track and research review and synthesis on effectiveness and safety track

These top two tracks of human research (Fig. 1) are usually important, including when, as often happens, new interventions are developed in an area where there are already existing interventions; this may be in order to gather cost-effectiveness data to inform policy on reimbursement decisions. With the development of a vaccine for COVID-19, as there are no existing alternatives, it is extremely unlikely that such steps would be necessary before regulatory approval and reimbursement decisions about the first approved vaccine. However, they might subsequently become very important if additional vaccines are developed and approved.

### Clinical and health service track and public policy and development track

#### Regulatory approval/first non-research use in patients and monitoring

This essential step has to occur after the safety, dosage and efficacy trials and, while there are calls for it to be completed as rapidly as possible, there are also pleas “*not to cut corners*” [30]. Jiang points out that vaccines for other major diseases “*have a long history of safe use and were developed in line with requirements of regulatory agencies*”*.* He has worked to develop vaccines for various coronaviruses since 2003 and describes some of the potential dangers [30]. Nevertheless, there is clearly considerable scope in the current situation for any vaccine for COVID-19 to jump, or even avoid entirely, the queues that often exist in the work of regulatory bodies. Various teams, including the Jenner Institute and Inovio, are talking about their candidate vaccine potentially being ready in later 2020 or early 2021. Some suggest it will first be made available using the speeded up or emergency use procedures that, as discussed above, the regulatory bodies are able to use in exceptional circumstances [23, 26, 33, 34]. A member of the Jenner Institute team referred to the critical importance of the principle of transparent informed consent [33]. Regulatory authorities are already beginning to work in parallel with the organisations developing candidate vaccines, so that they are fully aware of how the trials are progressing and might indicate the extent of the evidence that would be necessary for approval in this crisis situation, as noted previously with HIV/AIDS. Regulatory authorities will have crucial decisions to make in the coming months about how far they are willing to take greater risks about safety in relation to vaccine use as well as what greater risks might be taken with the health of volunteers in the trials (see above).

#### National policy announcement/guidelines advice

It is highly likely that such decisions on using the vaccine would already be in place when the vaccine was ready for use. Thus, whereas there can often be long delays before action is taken on this track to promote the use of an intervention, in this instance, such delays are extremely unlikely.

#### Reimbursement/financial support

It is to be expected that the vaccine manufacturers and healthcare systems will have worked out these arrangements before any vaccine receives regulatory approval. Thus, again, this sometimes lengthy process should be uncharacteristically unlikely to cause delays on this occasion. This is another clear example of where there are likely to be overlaps and work in parallel on many tracks. Various funding proposals are emerging and it is widely assumed that such will be the demand for a vaccine that more than one will go into mass production [34, 35]. Overlapping approaches being advocated and implemented include carrying out more research and development (R&D) through the public sector but bringing in the private sector primarily for manufacture and distribution and adopting ‘advance market commitments’ to incentivise private R&D; the first approach is being taken by the Jenner Institute. In the first few months, funding for the Jenner Institute’s COVID-19 research has come from various public and charitable sources, including the donations channelled through CEPI and direct funding from the United Kingdom public funders of health research [23, 27]. At the end of April 2020, the Jenner Institute partnered with the British–Swedish company AstraZeneca, with support from the British Government, for the development, manufacture and large-scale distribution of the vaccine on a not-for-profit basis, with only the costs of production and distribution being covered [35]. This approach is not completely novel in the vaccine space but is the largest of its kind to date – it will be interesting to see if such approaches spread to other areas of healthcare. The Moderna vaccine candidate also shows aspects of this extension of the public sector role with some of Moderna’s BARDA support being used to develop manufacturing capacity in the United States, through collaboration with Lonza US (part of Swiss-based Lonza), which will complement Moderna’s existing more limited United States manufacturing capacity. It is aiming to start production prior to the completion of trials and scaling global production to a billion doses per year [32].

Related to the second approach of ‘advance market commitments’, Silverman et al. describe how, in terms of increasing the speed of development of vaccines, the supply-side investments in R&D are the pressing issues but that getting the demand-side right is also important [36]. In contrast to the Jenner Institute model, they claim that if society wishes industry to invest up-front in the risky, high-cost business of vaccine development, it might need “*to offer the promise of a predictable market for an effective vaccine that offers both access for all and a reasonable return on investment – de-risking market (commercial) uncertainty while still expecting companies to absorb the scientific risk that their products will fail*”. They also illustrate the considerable commercial risks that companies have faced when developing vaccines, including against previous coronaviruses. In that context, they propose that the ‘market-driven value-based advanced commitment’ is the best option as it “*differentiates price according to efficacy, so incentivizing development and use of vaccines with higher rates of disease prevention*”, but could also ensure vaccines were available at manufacturing cost for low-income countries [36]. This illustrates how, in this extreme situation, ensuring a credible promise of sufficient resources being available is likely to play an important role across the various tracks in the development of a vaccine.

### Clinical practice track

Given the enormous health and economic burden being caused by COVID-19, it is reasonable to assume that, in contrast to what often happens [11, 14], there will be no delay from healthcare professionals in starting to give the vaccine to populations as soon as regulatory approval is secured and manufacturing capacity has geared up. CEPI has called for “*funding to support global COVID-19 vaccine development efforts guided by three imperatives: speed, manufacture and deployment at scale, and global access*” [23]. Questions about equity in deciding who receives the vaccines, and who receives them first, are of huge importance, but they are beyond the scope of this Commentary, which focuses on the time between starting research and the application of the intervention as a standard procedure within clinical practice in at least some countries. However, as noted above, some approaches can incentivise innovation while also encouraging a more equitable distribution than might otherwise occur.

## Lessons for speeding up future research

Currently, it appears as though progress through the tracks is likely to be rapid and several themes appear to be emerging. First, in every track, we can already see, or expect, the rapid progress that we saw examples of scattered among the various case studies described above but never saw concentrated in one case. Second, we can already see examples of additional acceleration activities speeding up processes in various tracks. The full lessons from the development of one or more vaccines against COVID-19 will, however, only become apparent after successful vaccine application globally to combat SARS-CoV-2. In the meantime, the analysis here should help to identify factors to monitor as it is apparent that the rapid speed on this occasion is likely to be caused by a combination of many factors. These might include some changes, such as improved research processes, which may be valuable in future for other streams of biomedical research. Our analysis could feed into debates that are already beginning about how the pandemic might influence research systems in the future, with an emphasis on reducing red tape in applications and a greater focus on societal impact [37].

At the other end of the spectrum, there will be changes that cannot be replicated, at least in such an extreme form. These include the significant increase and concentration of resources resulting from both the extra financing being mobilised because of the intense urgency of the crisis and the consensus on prioritisation of resources in terms of avoiding the queues for attention from decision-makers in the research and public policy systems. In between, there are many factors where the possibility of applying improvements in future will need careful scrutiny and perhaps promotion. These might include factors such as working at increased risk and working in parallel and cooperatively.

Gilbert is clear that cooperation is vital for tackling the current crisis, “*Work is continuing at a very fast pace, and I am in no doubt that we will see an unprecedented spirit of collaboration and cooperation, convened by WHO, as we move towards a shared global goal of COVID-19 prevention through vaccination*” [27]. The cooperation is even seeing old rivals, such as GlaxoSmithKline from the United Kingdom and Sanofi from France, forming a partnership to speed up progress by combining their complementary strengths, as part of wider cooperation in which vaccine developers such as GlaxoSmithKline are making their licenced adjuvants available for use with COVID-19 vaccines developed by others [23, 33]. How far such cooperation will extend into other areas of health and biomedical research is a crucial question, but it is clear that conventions around pharmaceutical intellectual property are being challenged in the COVID sphere [38]. There is increased attention on OpenIP as well as the Medicines for Malaria Venture, where intellectual property is retained but drugs are accessible to users in poorer countries at affordable prices [38]. Perhaps, going forward, there will be more support for such approaches as a way to speed up the development of interventions.

## Conclusions

It is claimed that, on average, it takes 17 years from starting research to its translation into products, policies, and practice [1] and the development of vaccines has been reported to take on average over 10 years [23, 24]. To measure such time-lags and identify ways of reducing them, in 2015, we published a matrix consisting of overlapping tracks in the translation from health research to products, policies and practice. In the current pandemic, it has proved useful to use the matrix to help interpret the progress of the enormous global effort to develop a vaccine against COVID-19, whilst also acknowledging that some revision to our approach could be useful. For example, the current situation highlights a fifth method of accelerating translation – the development of technologies, such as vaccine platforms, that allow a range of conditions to be rapidly addressed. It would be useful to consider how far such approaches might contribute to other areas.

Collectively, the vast global mobilisation is beginning to adopt each of the approaches noted as potential ways to reduce the time-lags. This is already being undertaken in the early overlapping tracks in the matrix, i.e. through the discovery (or basic) research and into the trial phases of human research. Additionally, there are already indications of how the approaches would be taken into the further overlapping tracks of human research and on to rapid decisions by regulatory and reimbursement bodies and, finally, into mainstream clinical practice. Reducing the time taken from the start of the research to its translation from the previous noted average of 17 years to anything like 17 months would be an astonishing achievement but not without costs.

If something went wrong with the vaccine used, then, as Jiang warned, there could be setbacks “*into the future*” [30]. Provided, however, that all the speed results in one or more safe and efficacious vaccines, there is a growing opinion that the pandemic might make medical science more nimble after the crisis has passed [22, 37] and result in greater preparedness in the future [21]. The situation is constantly evolving, with, for example, an update in *Nature* in mid-May 2020 including an account from Moderna that simultaneously reported the potentially promising preliminary findings from the Phase I trial, and an animal challenge study [39]. (This presentation of dual data is consistent with the general agreement at a virtual meeting of medicines regulatory authorities and WHO that, while prior to Phase I trials for a COVID-19 vaccine it was necessary to obtain some animal data, it was not always necessary to conduct animal challenge studies [40]). It would be highly desirable for a team of research analysts to monitor this unique situation prospectively to inform future discussions about the scope for realistically transferring lessons from the development of the COVID-19 vaccine into wider lessons for accelerating the translation of early research into health improvements.

## Data Availability

No datasets were generated during the production of this Commentary. The datasets generated and analysed during the original study are included in the original article [10].

## Abbreviations

CEPI: Coalition for Epidemic Preparedness Innovations
COVID-19: Coronavirus disease
NDA: New Drug Application
NIAID: National Institute of Allergy and Infectious Diseases
R&D: Research and development
RDS: Respiratory distress syndrome
